# Supplementary figures and images for: Infertility and fecundity loss of Wolbachia-infected Aedes aegypti hatched from quiescent eggs is expected to alter invasion dynamics
Source: PLoS Negl Trop Dis. 2021 Feb 16;15(2):e0009179. doi: 10.1371/journal.pntd.0009179 (PMC7909672; doi:10.1371/journal.pntd.0009179)

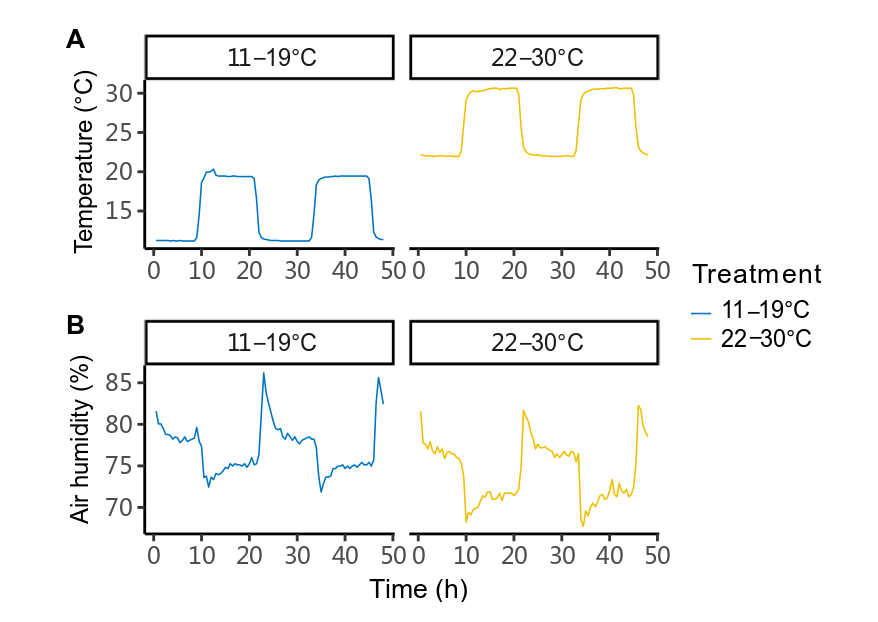

Supplement: S1 Fig — (A) Diurnal air temperature and (B) air humidity fluctuations in egg storage boxes. Incubators were set to temperature cycles of 11–19°C and 22–30°C. Data were measured by data loggers and shown across a representative 48 hour period to indicate the daily fluctuations during the experiment. (TIF) [file pntd.0009179.s001.tif]

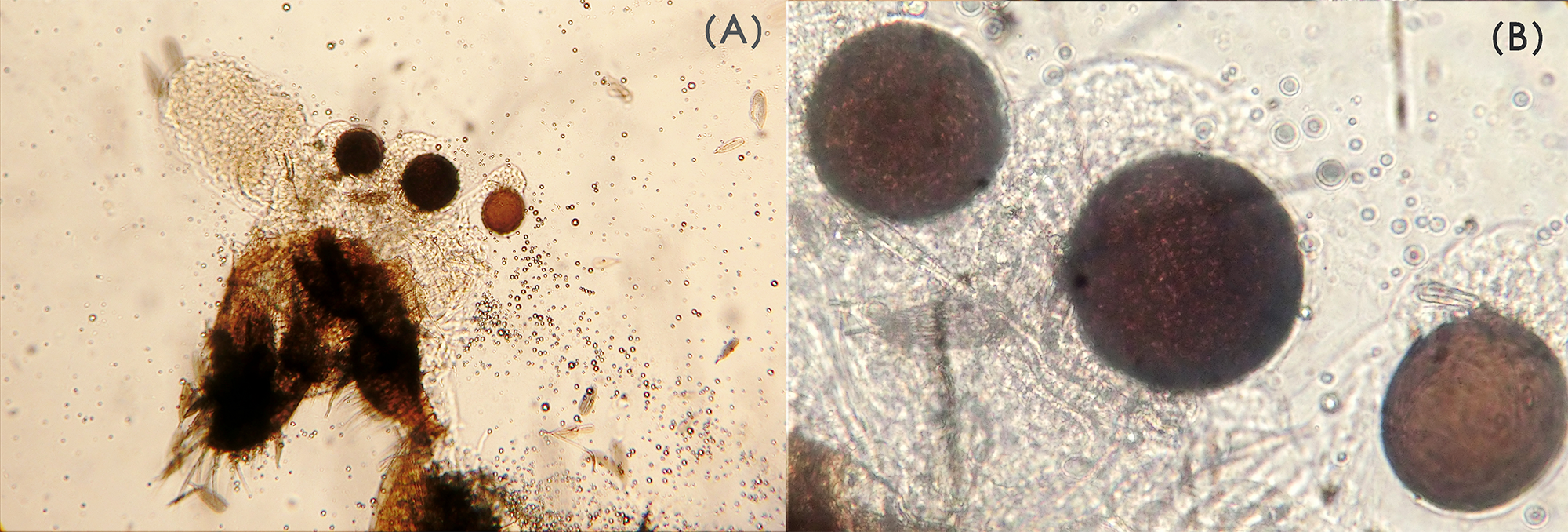

Supplement: S2 Fig — Three spermathecae of an infertile wAlbB-infected female Aedes aegypti were checked for insemination status under a (A) 40X objective lens or (B) 100X objective lens. The two on the left-hand side contain sperm, while the one on the right-hand side lacks sperm. (TIF) [file pntd.0009179.s002.tif]
